# Severe ectopic hidradenitis suppurativa refractory to adalimumab

**DOI:** 10.1016/j.jdcr.2026.08.039

**Published:** 2026-08-28

**Authors:** Ayushya Ajmani, Larissa Pastore, Heidi Bai, Shaofeng Yan, Natalie M. Fragoso

**Affiliations:** a Geisel School of Medicine at Dartmouth, Hanover, New Hampshire; b Department of Dermatology, Dartmouth Hitchcock Medical Center, Lebanon, New Hampshire; c Department of Pathology and Laboratory Medicine, Dartmouth Hitchcock Medical Center, Lebanon, New Hampshire

**Keywords:** ectopic HS, hidradenitis suppurativa, infliximab, TNF-α inhibitors

## Introduction

Hidradenitis suppurativa (HS) is a chronic inflammatory disease of pilosebaceous units characterized by recurrent nodules, abscesses, sinus tracts, and scarring in intertriginous regions.^1^ It affects women more frequently than men, with an annual incidence in the United States of 0.01% versus 0.005%, and is influenced by hormonal, genetic, and microbial factors.^1^ Pathogenesis involves follicular occlusion, dysbiosis, and innate immune dysregulation with upregulation of tumor necrosis factor-alpha, interleukin-1β, and interleukin-17 signaling pathways. Management of moderate-to-severe HS requires a multimodal approach including biologic therapies. Current guidelines recommend adalimumab as the first-line biologic, though some patients fail to achieve adequate response.^2^^,^^3^ Ectopic HS is an uncommon phenotype involving sites outside intertriginous regions, predominantly affecting men.^4^ Herein, we report a female patient with Hurley Stage III HS and body surface area (BSA) greater than 10% involving both intertriginous and ectopic sites, refractory to adalimumab but responsive to dose-escalated infliximab. This degree of widespread, multifocal disease has not been previously reported.

## Case report

A 25-year-old woman with a 10-year history of HS presented with progressive disease despite 5 years of treatment with adalimumab 40 mg weekly. Prior treatments included intralesional triamcinolone injections, doxycycline, and surgical excision of sinus tracts in the bilateral axilla. Prior history included severe nodulocystic acne treated with isotretinoin and prednisone. She denied history of joint pain and inflammatory bowel disease. Body mass index was 19.76 kg/m^2^.

Examination revealed plaques of inflammatory nodules, abscesses, draining sinus tracts, and extensive scarring affecting the face, abdomen, back, flanks, lower extremities, breasts, inframammary folds, axillae, and groin (Fig 1, *A*). BSA was 15%, and Hidradenitis Suppurativa Physician Global Assessment score was 5, indicating very severe disease. Punch biopsy from a leg plaque revealed dilated, keratin-plugged follicles, ruptured suppurative folliculitis, fibrosis, and granulomatous inflammation mixed with lymphocytes and abundant plasma cells, supporting the clinical impression of HS (Fig 2). Mycobacteria and fungi special stains were negative.

**Fig 1:**
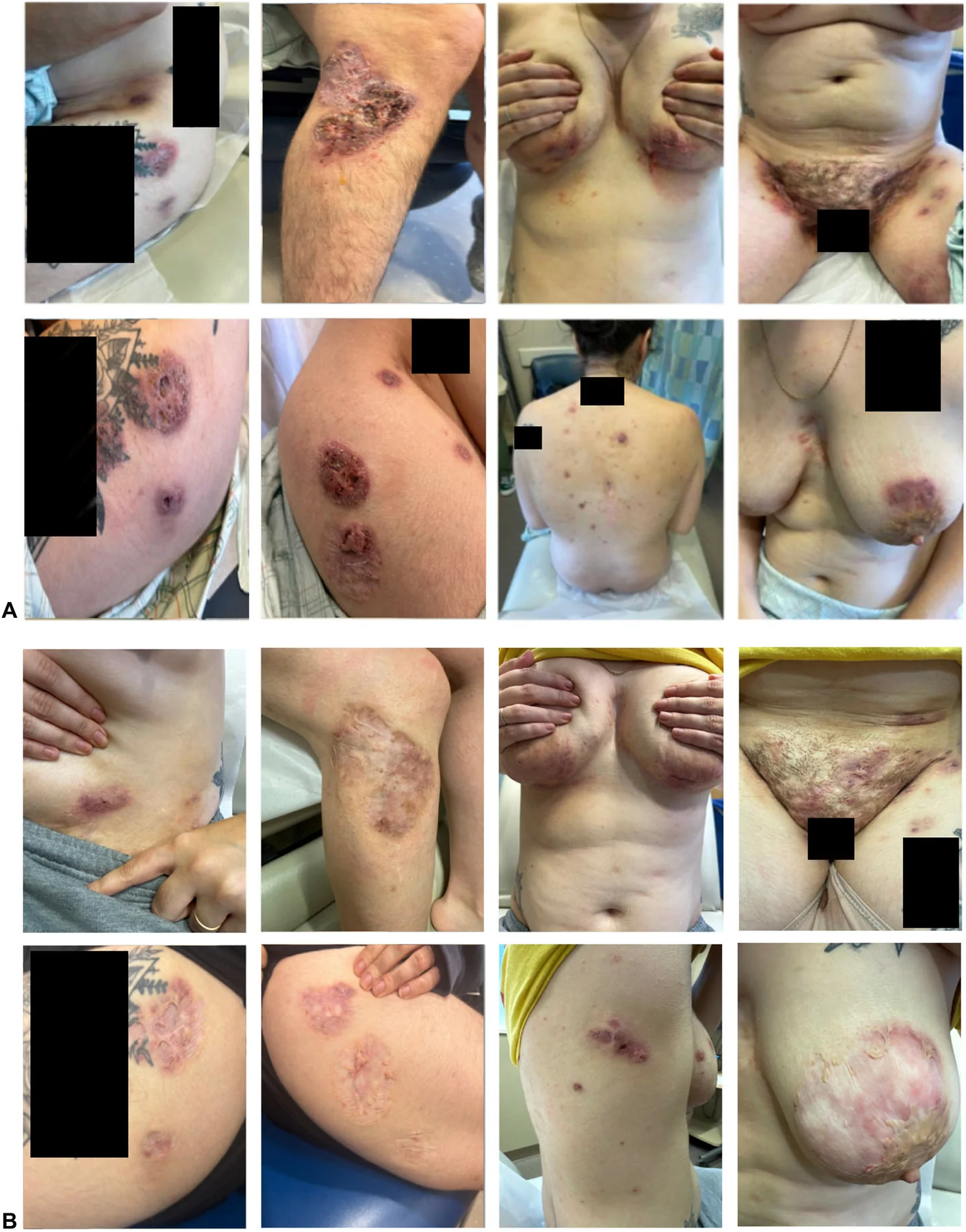
**A,** Baseline presentation showing widespread, multifocal hidradenitis suppurativa affecting the abdomen, groin, thighs, breasts, and lower extremities, characterized by active nodules, ulceration, and scarring. **B,** Follow-up after 12 months of infliximab demonstrating substantial reduction in erythema, induration, and active lesions across previously affected sites.

**Fig 2:**
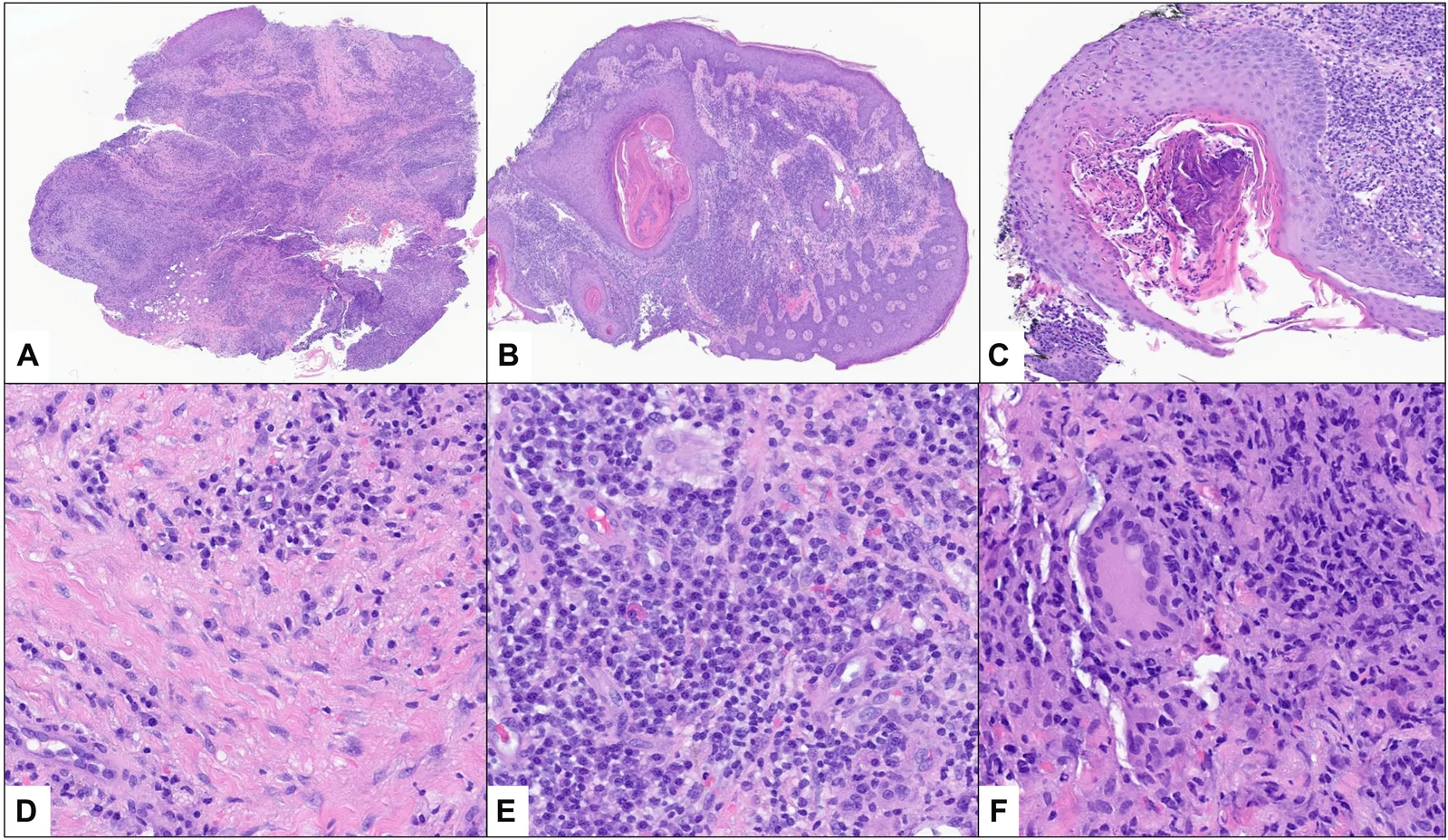
The biopsy findings: **A,** Dense inflammation (20×). **B,** Dilated and keratin-plugged follicles (20×). **C,** Ruptured suppurative folliculitis (100×). **D,** Dermal fibrosis (200×). **E,** Dense inflammation consisting of lymphocytes, abundant plasma cells, rare eosinophils (200×). **F,** Histiocytes and multinucleated giant cells (200×).

Adalimumab was discontinued. Infliximab was initiated at 7.5 mg/kg every 8 weeks and titrated to 10 mg/kg every 4 weeks. By the second infusion, drainage and lesion formation had markedly decreased (Fig 1, *B*), achieving an excellent clinical response after 12 months (BSA 3%, Hidradenitis Suppurativa Physician Global Assessment 2). Adjunctive management included intralesional steroids, topical resorcinol cream, sinus tract deroofing, CO_2_ laser treatment for scarring, and Alexandrite laser treatment for hair reduction.

## Discussion

To our knowledge, this is the first reported case of Hurley Stage III HS with greater than 10% BSA involvement at concurrent intertriginous and ectopic sites. Ectopic HS is an uncommon phenotype primarily affecting men, with a mean age at onset of approximately 40 years and a predilection for the head and neck, most often arising in the setting of established intertriginous disease.^2^^,^^3^ Our patient’s presentation is more diffuse and demographically distinct from the focal, later-onset, male-predominant cases described in prior literature. Notably, only 31% of ectopic HS patients have been reported to receive biologic therapy, underscoring the degree to which this phenotype remains underrecognized and undertreated.^4^

Diagnosis of ectopic HS can be challenging, as scarred nodules, plaques, and sinus tracts outside classic apocrine-bearing sites may mimic other follicular or neutrophilic dermatoses, including pyoderma gangrenosum (PG). Patients with other characteristic findings of PG, acne, and suppurative hidradenitis syndrome may also be difficult to diagnose. However, several features of this case are more consistent with a diagnosis of ectopic HS. The absence of undermined violaceous ulcers favored severe, multifocal HS. Our patient's biopsy showed dilated, keratin-plugged follicles with ruptured suppurative folliculitis, as is typically seen in HS.^5^^,^^6^ This follicular-centered pattern favors HS over the neutrophilic dermal infiltrate typical of PG. Ectopic HS lesions may also lack the paired comedones and interconnecting tracts typical of intertriginous disease, instead presenting as isolated nodules, cysts, or verrucous plaques best recognized alongside concurrent or antecedent classic disease.^5^ Further, her lesions arose alongside classic intertriginous distribution of HS, which is more suggestive of ectopic HS rather than PG or PG, acne, and suppurative hidradenitis syndrome.

Adalimumab, the first Food and Drug Administration-approved biologic for HS, achieved clinical response in approximately 50% of patients in phase III trials for classic intertriginous disease, with its efficacy in ectopic HS remaining largely undefined.^2^, ^3^, ^4^ Our patient’s 5-year treatment failure with adalimumab exemplifies this therapeutic challenge, particularly in the setting of extensive ectopic disease. Adalimumab trough levels and antidrug antibodies were not obtained during this patient’s treatment course; prospective assessment of these biomarkers may help guide treatment selection in future ectopic HS cases. Pooled data across 19 studies in classic HS report an overall infliximab response rate of 83%, with high-dose, high-frequency protocols consistently outperforming lower-dose regimens.^7^^,^^8^ However, data specific to infliximab use in ectopic HS remain sparse, with only 1 prior case of successful treatment reported in the literature; our patient represents the second such case.^8^

In our patient, initiation of infliximab at 7.5 mg/kg and escalation to 10 mg/kg every 4 weeks yielded an 80% BSA reduction and Hidradenitis Suppurativa Physician Global Assessment score improvement from 5 (very severe disease) to 2 (mild disease) at 12 months, demonstrating that meaningful, sustained response to dose-escalated infliximab is achievable in severe ectopic disease following adalimumab failure. Metabolic factors, including obesity, are recognized contributors to HS severity; however, this patient was neither obese nor diabetic, and no concurrent weight-loss or glycemic intervention accompanied the switch to infliximab. These features argue against a substantial metabolic contributor to her improvement.

Management of ectopic HS in multiple locations can be challenging. Beyond inhibition of tumor necrosis factor-a, interleukin-17A inhibition (secukinumab) and Janus kinase inhibition (upadacitinib) have shown efficacy in refractory HS, though data specific to ectopic disease remain limited to isolated reports.^9^^,^^10^ The use of adjunctive interventions in this patient reflects the multimodal approach required for optimal management of severe HS, including ectopic disease.

This case broadens the clinical spectrum of HS and supports the use of high-frequency, high-dose infliximab as a viable option in severe, refractory ectopic disease following anti-tumor necrosis factor-α failure. Future prospective studies should examine treatment regimens specific to ectopic HS, including biomarker-guided biologic selection, to better inform clinical guidelines.

### Declaration of generative AI and AI-assisted technologies in the writing process

Not used.

## Conflicts of interest

Dr Fragoso has served as a consultant for UCB and as an investigator for Abbvie, Amgen, Acelyrin, Boehringer Ingelheim, Insmed, Takeda, and Target Derm. Drs Pastore, Bai, Yan, and Author Ajmani have no conflicts of interest to declare.
